# Mineral-Enriched Deep-Sea Water Modulates Lactate Metabolism via PGC-1α-Mediated Metabolic Reprogramming

**DOI:** 10.3390/md17110611

**Published:** 2019-10-27

**Authors:** Byung Geun Ha, Sung Suk Jung, You Kyung Jang, Byong Yeob Jeon, Yun Hee Shon

**Affiliations:** 1 Biomedical Research Institute, Kyungpook National University Hospital, 50 Samduk 2ga Jung-gu, Daegu 41944, Korea; hbgbio@naver.com (B.G.H.); xjworld1180@naver.com (S.S.J.); 2 QBM Co., Ltd., Seoul 06752, Korea; ykjang01@qbm.co.kr (Y.K.J.); byjeon01@qbm.co.kr (B.Y.J.)

**Keywords:** C2C12 myotubes, deep-sea water, lactate metabolism, metabolic disorders, mineral deficiency, PGC-1α

## Abstract

Metabolic disorders such as diabetes and obesity are serious global health issues. These diseases are accelerated by mineral deficiencies, emphasizing the importance of addressing these deficiencies in disease management plans. Lactate metabolism is fundamentally linked to glucose metabolism, and several clinical studies have reported that blood lactate levels are higher in obese and diabetic patients than in healthy subjects. Balanced deep-sea water contains various minerals and exhibits antiobesity and antidiabetic activities in mice; however, the impact of balanced deep-sea water on lactate metabolism is unclear. Thus, we evaluated the effects of balanced deep-sea water on lactate metabolism in C2C12 myotubes, and found that balanced deep-sea water mediated lactate metabolism by regulating the gene expression levels of lactate dehydrogenases A and B, a monocarboxylate transporter, and a mitochondrial pyruvate carrier. The activities of peroxisome proliferator-activated receptor gamma coactivator-1α (PGC-1α) and signaling molecules involved in PGC-1α activation were also upregulated by treatment with balanced deep-sea water. These results suggest that balanced deep-sea water, which can mediate lactate metabolism, may be used to prevent or treat obesity and diabetes mellitus.

## 1. Introduction

Dysregulation of lactate metabolism is related to the development of metabolic disorders, including obesity and diabetes mellitus. Glucose and alanine, the primary sources of intracellular lactate in humans, are converted to pyruvate, which is subsequently reduced to lactate by lactate dehydrogenase (LDH) in the cytoplasm. The A and B subunits of LDH mediate the bidirectional conversion of lactate and pyruvate. The M isoform of LDH (LDH A) produces lactate from pyruvate in skeletal muscles and other highly glycolytic tissues, while the H isoform (LDH B) functions in the heart and promotes lactate metabolism via oxidation to produce pyruvate, enabling cells to use lactate as a nutrient for oxidative metabolism [1].

Lactate is transported across the cell membrane by monocarboxylate transporters (MCTs), four of which (MCT1, MCT2, MCT3, and MCT4) have been reported to date [2]. MCTs mediate the bidirectional cotransport of protons and substrates such as lactate, pyruvate, and fatty acids across the plasma membrane; in particular, MCT4 and MCT1 are responsible for lactate export and import, respectively [3].

Pyruvate is a major substrate for oxidative metabolism and an essential branching point for the synthesis of glucose, lactic acid, fatty acids, cholesterol, and amino acids. In humans, mitochondrial pyruvate carrier 1 (MPC1) and MPC2 form a hetero-oligomeric complex in the inner mitochondrial membrane to facilitate pyruvate transport [4].

A previous study has shown that peroxisome proliferator-activated receptor gamma coactivator-1α (PGC-1α) induces a transcription program that promotes lactate metabolism and mediates skeletal muscle adaptations, which improve metabolic health [5]. In skeletal muscles, PGC-1α induces *LDH B* expression via estrogen-related receptor-α (*ERR-α*) and suppresses the *LDH A* transcription by promoting the expression of the retinoid X receptor (*RXR*) gene, which also attenuates *Myc* expression. Therefore, PGC-1α controls the configuration of the LDH complex and suppresses the increase in blood lactate levels during metabolic disease [6].

AMP-activated protein kinase (AMPK) is implicated in the therapeutic effects of metformin [7], thiazolidinediones [8], and exercise [9], all of which form the foundation of clinical care for diabetes mellitus and related metabolic diseases. AMPK plays an important role in initiating mitochondrial biosynthesis to help the body cope with energy deficiencies and regulate mitochondrial gene expression in the striated muscle [10]. In particular, the activation of the AMPK-enhanced NAD-dependent deacetylase sirtuin-1 (SIRT1) results in the positive regulation of downstream targets, including PGC-1α [11]. Moreover, AMPK and SIRT1 activation can modulate the expression of genes related to energy metabolism and increase the *PGC-1α* gene expression or protein activity through a change in the acetylation state.

Mineral deficiencies are very common in patients with diabetes mellitus, obesity, hypertension, and cardiovascular disease. These deficiencies significantly influence the onset of metabolic disorders and overall health; thus, the optimal intake of minerals can potentially fine-tune the metabolism and improve patient health. Deep-sea water (DSW) has been formed over millennia and is a sustainable natural marine resource that is rich in essential minerals, including magnesium, calcium, and sodium, as well as microelements, such as iron, manganese, and selenium. DSW has been reported to modulate hematopoiesis [12], reduce serum cholesterol levels [12], heal atopic dermatitis [13], and alleviate arteriosclerosis [14]. 

Our recent studies have shown that balanced DSW (BDSW) shows antidiabetic activity, which reduces the fasting blood glucose level and improves impaired glucose tolerance in type-1 [15] and type-2 [16] diabetes mouse models. Additionally, BDSW exhibits antilipidemic activity, inhibiting adipocyte hypertrophy and visceral fat accumulation, and reduces liver steatosis by regulating lipid metabolism [17]. Moreover, we have previously found that BDSW improved diabetes mellitus and obesity by regulating the mitochondrial metabolism [18,19]. In this study, we evaluated the effects of BDSW on lactate metabolism in myotubes.

## 2. Results

### 2.1. BDSW Cytotoxicity 

**Figure 1:**
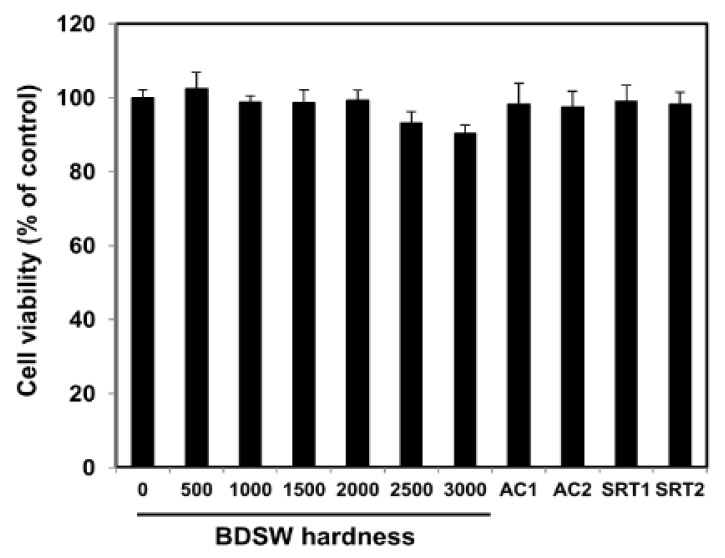
Effect of balanced deep-sea water (BDSW) on cell viability of C2C12 myotubes. Cells were incubated with BDSW of various hardness levels (0–3000), 0.25 mM 5-aminoimidazole-4-carboxamide ribonucleotide (AICAR; AC1), 0.5 mM AICAR (AC2), 5 μM SRT1720 (SRT1), or 10 μM SRT1720 (SRT2) for 12 h. The data represent the mean ± standard error of the mean (SEM) of three independent experiments.

The cytotoxicity of BDSW was evaluated by measuring the viability of mouse C2C12 myotube cells. BDSW did not show significant cytotoxic effects for C2C12 cells at hardness levels of up to 3000 (Figure 1). Because of the lack of a measurable effect of water hardness on cell viability, no further morphological analyses of the cells were conducted.

### 2.2. BDSW Modulated Expression of Lactate Production-Related Genes 

**Figure 2:**
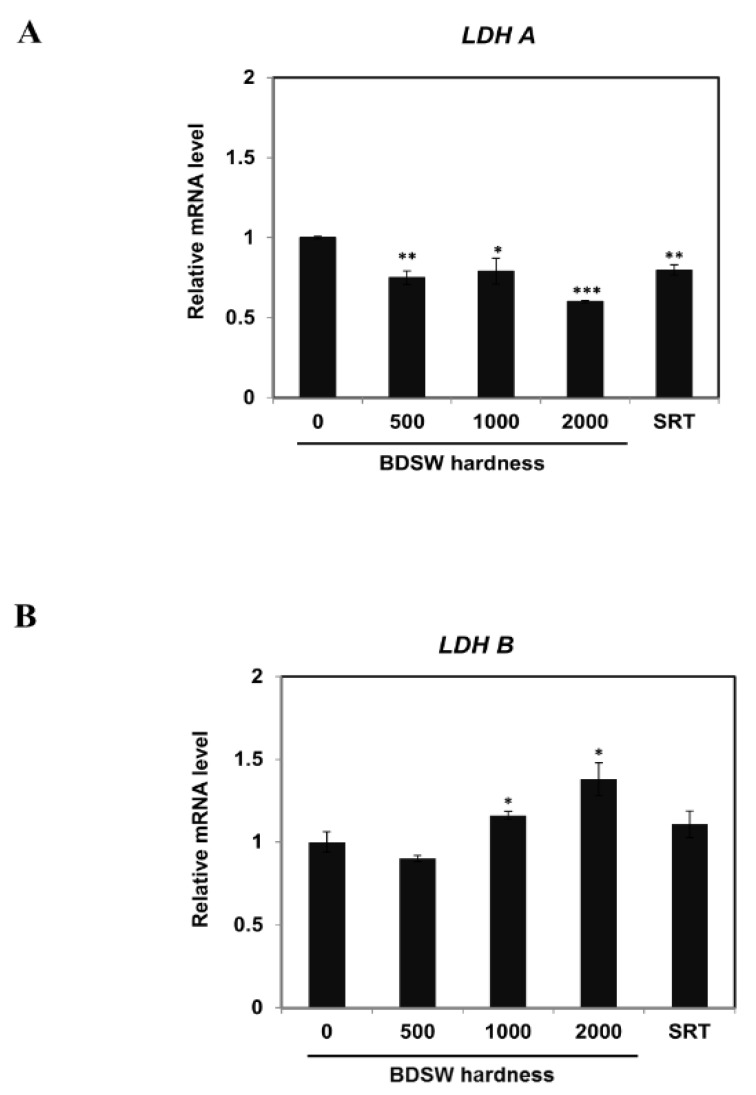
Effects of BDSW on the expression of genes related to lactate production in C2C12 myotubes. Cells were incubated with BDSW of various hardness levels (0, 500, 1000, or 2000) or 10 μM SRT1720 (SRT) for 12 h. The mRNA levels of (**A**) *LDH A* and (**B**) *LDH B* represent the mean ± SEM of three independent experiments. *p < 0.05; **p < 0.01; ***p < 0.001 vs. untreated control.

To evaluate the effects of BDSW on lactate production, we examined the mRNA expression of *LDH A* and *LDH B* in C2C12 myotubes. BDSW decreased the *LDH A* mRNA expression in a hardness-dependent manner (Figure 2A), whereas the *LDH B* mRNA expression gradually increased as the BDSW hardness increased (Figure 2B). These results suggest that BDSW can modulate lactate production.

### 2.3. BDSW Enhanced PGC-1α Expression and Activity in C2C12 Myotubes 

**Figure 3:**
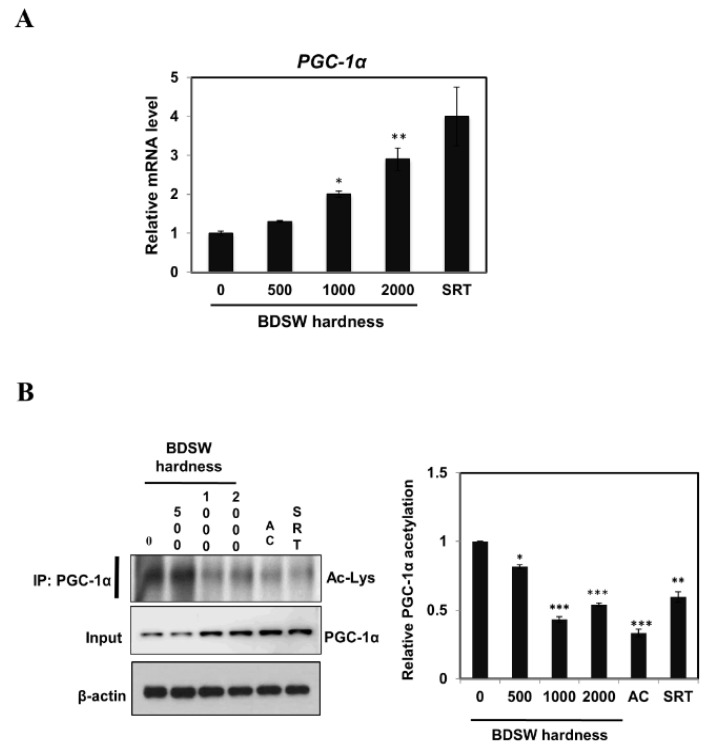
Effects of BDSW on *PGC-1α* gene expression (**A**) and protein activity (**B**) in C2C12 myotubes. (**A**) Relative *PGC-1α* mRNA levels in C2C12 myotubes incubated with BDSW of various hardness levels (0, 500, 1000, or 2000) or 10 μM SRT1720 (SRT) for 12 h. The data represent the mean ± SEM of three independent experiments. *p < 0.05; **p < 0.01 vs. untreated control. (**B**) Representative western blots and relative quantification of the acetylated versus total PGC-1α protein. Cells were incubated with BDSW of various hardness levels (0, 500, 1000, or 2000), 0.5 mM AICAR (AC), or 10 μM SRT1720 (SRT) for 1 h. PGC-1α was immunoprecipitated from nuclear extracts and immunoblotted using an anti-acetylated lysine (Ac-Lys) antibody to measure the acetylated protein or an anti-PGC-1α antibody to determine the total protein. IP, immunoprecipitation. The data represent the mean ± SEM of three independent experiments. *p < 0.05; **p < 0.01; ***p < 0.001 vs. untreated control.

To study the effects of BDSW on lactate homeostasis, we measured PGC-1a expression and activity in C2C12 myotubes. BDSW treatment of C2C12 myotubes increased the *PGC-1α* mRNA levels in a hardness-dependent manner (Figure 3A) and decreased PGC-1α acetylation (Figure 3B). These results suggest that BDSW treatment can upregulate the *PGC-1α* gene expression and protein deacetylation, which significantly promotes the PGC1-α activity.

### 2.4. Potential Mechanisms of BDSW Effects on Lactate Metabolism 

**Figure 4:**
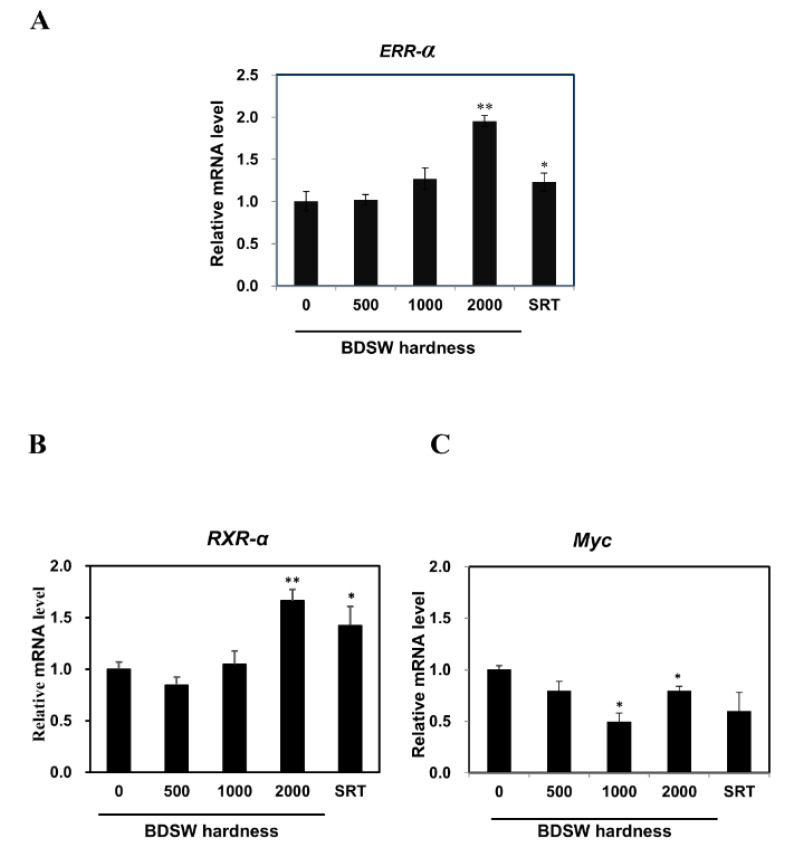
Effects of BDSW on the expression of genes regulating *LDH B* and *LDH A* expression in C2C12 myotubes. Cells were incubated with BDSW of various hardness levels (0, 500, 1000, or 2000) or 10 μM SRT1720 (SRT) for 12 h. The relative mRNA expression levels of (**A**) *ERR-α* were associated with *LDHB* expression, while those of (**B**) *RXR-α* and (**C**) *Myc* were associated with *LDHA* expression, as determined by qRT-PCR. The data represent the mean ± SEM of three independent experiments. *p < 0.05; **p < 0.01 vs. untreated control.

**Figure 5:**
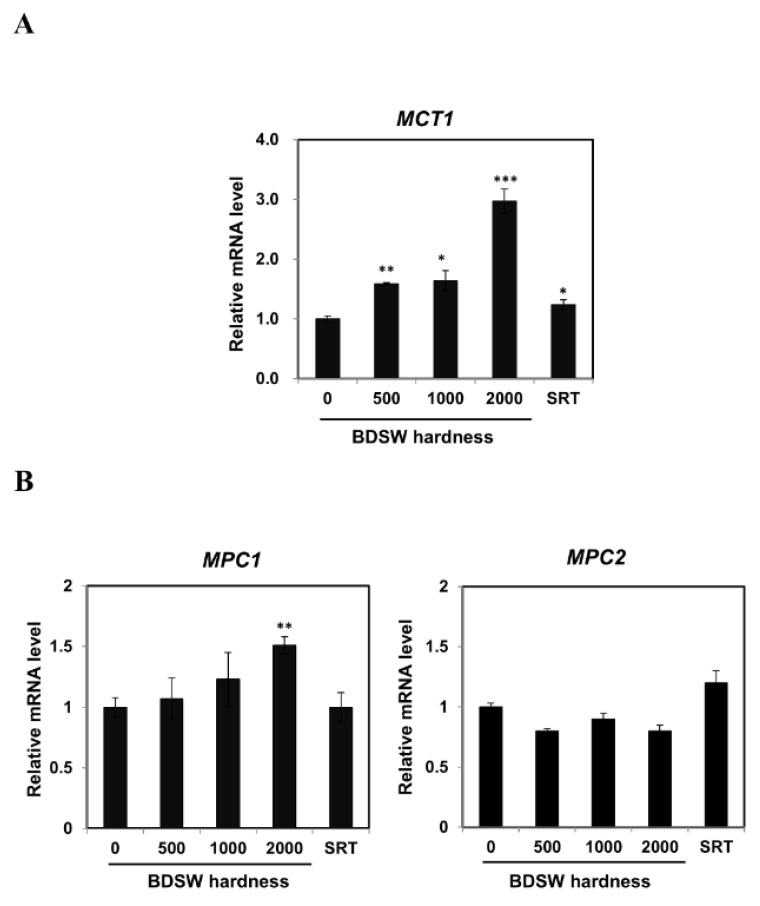
Effects of BDSW on the expression of genes related to lactate (*MCT1*) and pyruvate (*MPC1* and *MPC2*) transport in C2C12 myotubes. Cells were incubated with BDSW of various hardness levels (0, 500, 1000, or 2000) or 10 μM SRT1720 (SRT) for 12 h. The relative expression levels of (**A**) *MCT1* and (**B**) *MPC1* and *MPC2* represent the mean ± SEM of three independent experiments. *p < 0.05; **p < 0.01; ***p < 0.001 vs. untreated control.

To clarify the molecular mechanism involved in the mediation of lactate production by BDSW, we used quantitative real-time polymerase chain reaction (qRT-PCR) to examine the expression of genes encoding regulators of *LDH B* and *LDH A* in C2C12 myotubes. We found that BDSW treatment increased the *ERR-α* and *RXR-α* expression (Figure 4A,B) and decreased that of *Myc* (Figure 4C). These results suggested that BDSW could modulate lactate production by reprogramming genes that regulate *LDH A* and *LDH B* expression. To assess the effects of BDSW on the transport of lactate and pyruvate, we examined *MCT1*, *MPC1,* and *MPC2* mRNA levels in C2C12 myotubes and observed an increase in *MCT1* (Figure 5A) and *MPC1* (Figure 5B) expression upon an increase in BDSW hardness.

### 2.5. Signaling Molecules Involved in PGC-1α Activation 

The AMPK-mediated increase in SIRT1 activity regulates the activity of downstream SIRT1 targets (e.g., PGC-1α) [11]. Through these pathways, PGC-1α induces transcription programs that enhance lactate metabolism and mediate skeletal muscle adaptation to improve metabolic health [6,11]. AMPK is activated through its phosphorylation by AMPK kinases, liver kinase B1, and Ca^2+^/calmodulin-dependent protein kinase II in response to an increase in the AMP:ATP ratio. Phosphorylated AMPK is critical in skeletal tissues.

**Figure 6:**
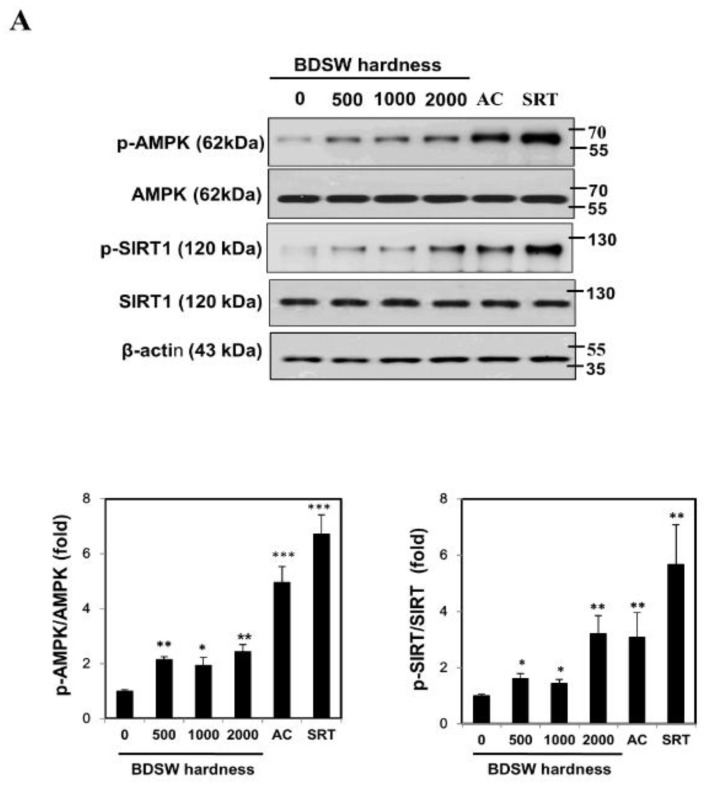

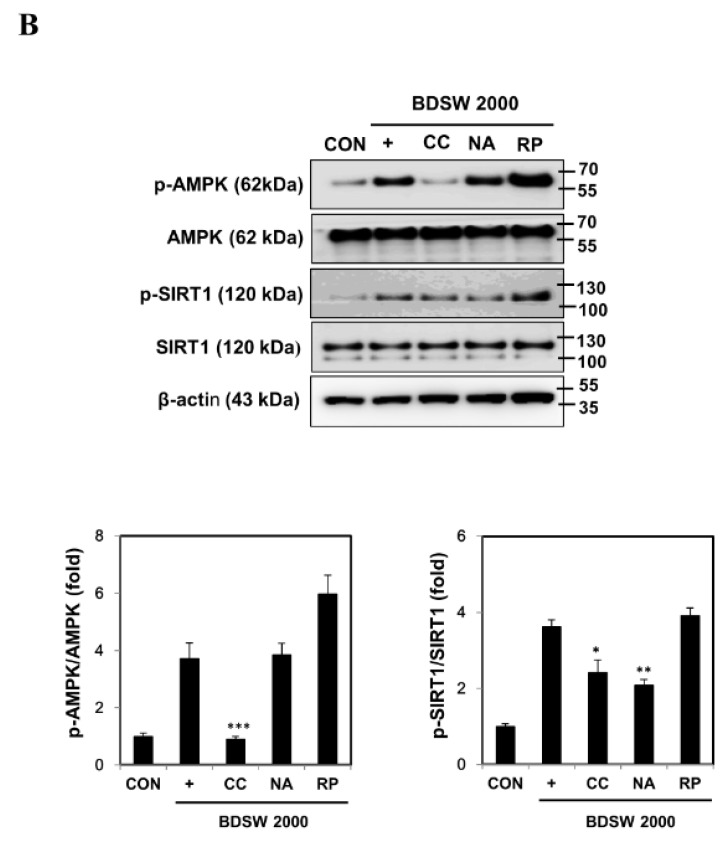
Effects of BDSW, with and without kinase inhibitors, on the activities of signaling molecules involved in lactate metabolism in C2C12 myotubes. (**A**) Representative western blots and quantitative analysis of phospho-AMPK (p-AMPK), total AMPK, p-SIRT1, and total SIRT1 levels in C2C12 myotube lysates following treatment with BDSW of various hardness levels (0, 500, 1000, or 2000), 0.5 mM AICAR (AC), or 10 µM SRT1720 (SRT) for 1 h. β-Actin was used as a loading control. The data represent protein levels relative to those of β-actin and are presented as the mean ± SEM. *p < 0.05, **p < 0.01, and ***p < 0.001 vs. untreated control. (**B**) Representative western blots and quantitative analysis of p-AMPK, total AMPK, p-SIRT1, and total SIRT1 levels in C2C12 myotubes treated with 0.5 μM compound C (CC), 1 mM nicotinamide (NA), or 100 nM rapamycin (RP) for 0.5 h, followed by incubation with BDSW with a hardness of 2000 for 1 h. β-Actin was used as a loading control. CON, control. The data represent protein levels relative to those of β-actin and are presented as the mean ± SEM. *p < 0.05; **p < 0.01; ***p < 0.001 vs. CON.

In light of these findings, we next studied the effects of BDSW treatment on signaling molecules involved in PGC-1α activation in C2C12 myotubes. We found that BDSW treatment augmented the AMPK and SIRT1 phosphorylation (Figure 6A). These results suggested that BDSW could control lactate homeostasis via PGC-1α activation by enhancing AMPK and SIRT1 activity.

To confirm the effect of BDSW treatment on signaling molecules involved in PGC-1α activation, we investigated the effects of inhibitory substances, Compound C (CC; an ATP-competitive repressor of AMPK), nicotinamide (NA; a SIRT1 repressor), and rapamycin [RP; a mammalian target of rapamycin (mTOR) repressor], on the phosphorylation of AMPK and SIRT1. BDSW-induced AMPK and SIRT1 phosphorylation was reduced following treatment with CC and NA, respectively, but not with RP (Figure 6B).

## 3. Discussion

Lactic acid metabolism is fundamentally linked to glucose metabolism; therefore, metabolic disorders that affect glucose metabolism, such as obesity and diabetes, alter lactic acid homeostasis. Additionally, fasting plasma lactate levels are higher in obese and diabetic patients compared to those in healthy individuals [20,21,22], and the release of lactate by muscle preparation from obese patients with and without diabetes mellitus is significantly higher than that observed in the control [23]. However, there have been no in vitro studies investigating the mechanism of regulation of lactate metabolism in diabetes and obesity. 

**Figure 7:**
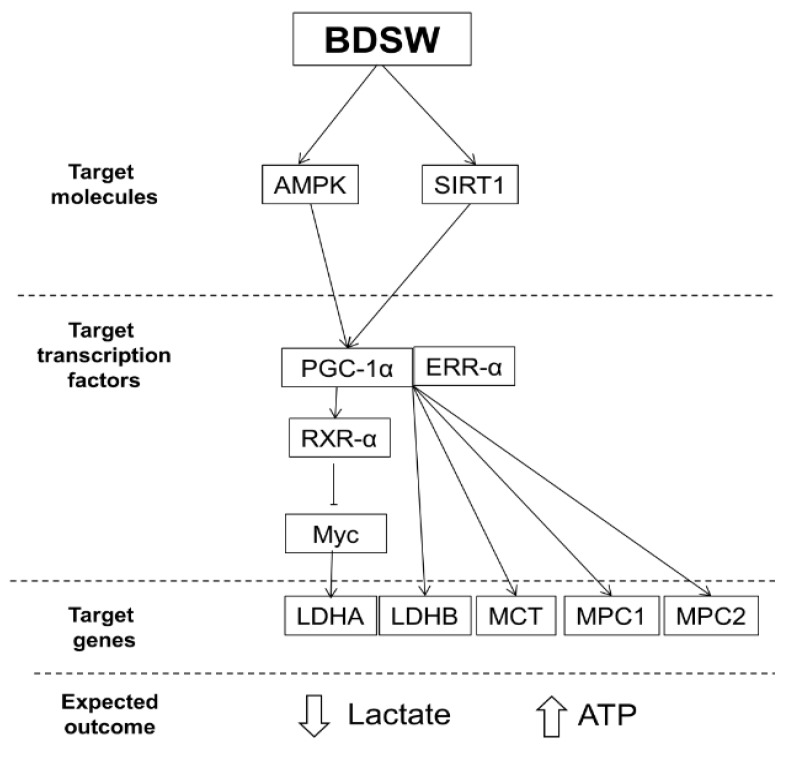
Proposed mechanism for modulation of lactate metabolism by BDSW in C2C12 myotubes. BDSW, balanced deep-sea water; AMPK, AMP-activated protein kinase; SIRT1, NAD-dependent deacetylase sirtuin-1; PGC-1α, peroxisome proliferator-activated receptor gamma coactivator-1α; ERR-α, estrogen-related receptor-α; RXR-α, retinoid X receptor-α; MCT, monocarboxylate transporter; and MPC, mitochondrial pyruvate carrier.

In this study, we evaluated the effects of BDSW, which shows antidiabetic and antilipidemic activities, on lactate metabolism in myotubes. Our results showed that BDSW mediated lactate metabolism in C2C12 myotubes by regulating the expression of *LDH A*, *LDH B*, *MCT1*, and *MPC1,* and also increased the activities of PGC-1α and its associated signaling molecules (Figure 7). We have previously shown that BDSW inhibits adipocyte hypertrophy and hepatic steatosis in a high-fat diet (HFD)-induced obesity mouse model [17]. BDSW ameliorated glycometabolism in HFD-induced type-2 diabetic mice and streptozotocin-induced type-1 diabetic mice [15,16]. Additionally, BDSW upregulated mitochondrial biogenesis and function in muscle tissues from obese mice, 3T3-L1 preadipocytes, and myotube cells [18,19]. Our findings, showing that BDSW improved lactate metabolism in C2C12 myotubes, suggest that BDSW can beneficially affect the onset and progression of obesity and diabetes, although this hypothesis requires further study. 

We hypothesized that BDSW could promote metabolic adaptation in skeletal muscles through PGC-1α activation. Our study showed that PGC-1α induced the *LDH B* expression in C2C12 myotubes following BDSW treatment. Furthermore, PGC-1α activity reduced the expression of *LDH A*, as well as that of one of its regulators (*Myc*). These results indicated that changes in PGC-1α expression could modulate the composition of the LDH complex, suggesting that BDSW can affect lactate production via PGC-1α activation in C2C12 myotubes. Our findings suggest that PGC-1α activity regulates lactate homeostasis in C2C12 myotubes in response to BDSW treatment, thus improving the metabolic health. Although we did not measure lactate and ATP in C2C12 myotubes or in the culture medium, we expect that the lactate content would decrease and that of ATP would increase in BDSW-treated C2C12 myotubes (Figure 7).

In conclusion, the described regulatory effects of BDSW on the lactate metabolism provide an insight into the mechanism of the antidiabetic and antiobesity activities of BDSW. However, further studies in animal models and through clinical trials are required to fully elucidate how minerals in the form of BDSW can be used in the prevention and treatment of diabetes mellitus and obesity.

## 4. Materials and Methods 

### 4.1. Materials 

Immortalized mouse myoblast C2C12 cells (CRL-1772) were purchased from the American Type Culture Collection (Manassas, VA, USA). 5-Aminoimidazole-4-carboxamide ribonucleotide (AICAR), 3-(4,5-dimethyl-2-thiazolyl)-2,5-diphenyl-2H-tetrazolium bromide (MTT), dimethyl sulfoxide (DMSO), 6-[4-(2-piperidin-1-ylethoxy)phenyl]-3-pyridin-4-ylpyrazolo[1,5-a]pyrimidine (CC), NA, and RP were obtained from Sigma–Aldrich (St. Louis, MO, USA). Mouse antihuman AMPK (#2793), rabbit antihuman phospho-AMPK (#2535), and rabbit antihuman phospho-SIRT1 (#2314) were purchased from Cell Signaling Technology (Danvers, MA, USA). SRT1720, protein A/G agarose, rabbit antihuman SIRT1 (Sc-15404), rabbit antihuman PGC-1α (Sc-13067), anti-acetylated lysine (Ac-Lys; Sc-32268), mouse antihuman β-actin (Sc-47778), goat antimouse IgG–horseradish peroxidase (HRP; Sc-2005), and goat antirabbit IgG-HRP (Sc-2004) were obtained from Santa Cruz Biotechnology (Dallas, TX, USA). The enhanced chemiluminescence (ECL) Plus western blotting substrate was purchased from Pierce Biotechnology (Rockford, IL, USA). TRIzol was purchased from Invitrogen Life Technologies (Carlsbad, CA, USA). The PrimeScript first-strand cDNA synthesis kit was purchased from Takara Bio (Shiga, Japan). FastStart universal SYBR Green master was obtained from Roche Applied Science (Basel, Switzerland).

### 4.2. BDSW Preparation

DSW was pumped at a depth of 0.5 km from a region located 6.7 km from the shoreline of Oho-Ri, Goseong (38.200° N and 128.340° E, East Sea, Korea), as previously described [13]. To eliminate marine microorganisms, DSW was filtered through a microfiltration membrane (Synopex, Pohang, Korea). After filtration, reverse osmosis was used to separate DSW minerals from desalted water (Vontron Technology Co., Ltd., Beijing, China). Extracted minerals (Mg and Ca) were combined with desalted DSW at a 3:1 ratio (Mg to Ca) to obtain BDSW. Mg and Ca contents of the BDSW were measured using a Dionex ICS-1100 basic integrated ion chromatography system (Thermo Fisher Scientific, Sunnyvale, CA, USA). The hardness of BDSW was determined based on the Mg and Ca concentrations as follows: hardness = Mg (mg/L) × 4.1 + Ca (mg/L) × 2.5. The mineral content of the BDSW at a hardness of 2000 is shown in Table 1.

### 4.3. C2C12 Cell Culture and Differentiation

C2C12 cells were maintained in Dulbecco’s modified Eagle’s medium (DMEM; Hyclone Laboratories, Inc., South Logan, UT, USA) supplemented with 10% fetal bovine serum (FBS; Hyclone Laboratories, Inc.), 1% penicillin, and 1% streptomycin at 37 °C in an incubator with 5% CO_2_. Cells were grown to confluence, and differentiation was induced by replacing 10% FBS with 2% horse serum. The medium was changed daily, and differentiated C2C12 myotubes were used 4–5 days after plating.

### 4.4. BDSW Treatment

BDSW or desalted water was used to dissolve the DMEM powder. DMEM containing BDSW was diluted with DMEM containing desalted water to prepare BDSW of various hardness levels (500, 1000, 1500, 2000, 2500, or 3000). DMEM prepared with desalted water was used as a control. C2C12 myotubes were exposed to BDSW of various hardness levels or to AICAR (0.5 mM) or SRT1720 (10 μM) as positive controls for 1 or 12 h.

### 4.5. Inhibitor Treatments

C2C12 myotubes were incubated for 30 min with or without 0.5 μM CC (AMPK phosphorylation inhibitor), 1 mM NA (SIRT1 phosphorylation inhibitor), or 100 nM RP (mTOR phosphorylation inhibitor), followed by incubation with BDSW at a hardness of 2000 for 1 h.

### 4.6. Cell Toxicity

C2C12 myotubes were plated into a 96-well culture plate and exposed to BDSW of various hardness levels (0, 500, 1000, 1500, 2000, 2500, or 3000), AICAR (0.25 mM or 0.5 mM), or SRT1720 (5 μM or 10 μM) for 12 h. The culture media were replaced with a medium containing MTT (5 mg/mL), and the plate was incubated in the dark for 4 h. DMSO was used to dissolve the formazan crystals formed. Absorbance was measured at 570 nm using a microplate reader (Molecular Devices, Sunnyvale, CA, USA).

### 4.7. qRT-PCR

Total RNA was isolated from C2C12 myotubes using TRIzol (Invitrogen), as described previously [19]. The PrimeScript kit was used to synthesize cDNA from RNA following the manufacturer’s protocol. qRT-PCR was performed in triplicate using the FastStart SYBR Green master mix and a CFX96 Touch RT-PCR system (Bio-Rad Laboratories, Hercules, CA, USA). Gene expression levels were normalized to those of β-actin (set to 1.0), and relative expression was calculated using the 2^−∆∆Ct^ method. Table 2 shows the sequences of the primers that were used for these experiments.

### 4.8. Western Blot Analysis

Cells were washed with Tris-buffered saline and lysed in radioimmunoprecipitation assay buffer (Thermo Fisher Scientific). Proteins were separated by sodium dodecyl sulfate polyacrylamide gel electrophoresis and then transferred to nitrocellulose membranes (Whatman GmbH, Dassel, Germany), which were incubated with 5% skim milk at 25 °C for 1 h. Subsequently, the membranes were incubated with primary antibodies (1:1000) at 4 °C overnight, followed by incubation with secondary antibodies (1:3000) for 1 h on a shaker. Bands were visualized using the ECL western blot substrate, and band intensities were quantified using the ImageJ software v1.51j (National Institutes of Health, Bethesda, MD, USA).

### 4.9. Immunoprecipitation

Immunoprecipitation was performed using a nuclear complex Co-IP kit (Active Motif, Carlsbad, CA, USA) to analyze the PGC-1α activity. For co-immunoprecipitation, nuclear protein extracts were incubated with agitation at 4 °C overnight with diluted anti-PGC-1α and then absorbed onto protein G agarose beads at 4 °C for 2 h. The immunoprecipitates used for immunoblotting were obtained with either anti-PGC-1α or anti-Ac-Lys. Immunoreactive bands were detected by ECL, and band intensities were measured using the ImageJ software.

### 4.10. Statistical Analysis

Data were analyzed by one-way analysis of variance, followed by Dunnett’s test, using the SPSS Statistics software v.11.0 (SPSS, Inc., Chicago, IL, USA). Statistical significance was considered at a *p*-value < 0.05.

## Figures and Tables

**Table 1:** Mineral content of balanced deep-sea water (BDSW) at a hardness of 2000 used in this study.

Mineral	BDSW (mg/L)	Mass Concentration (mg/L)	Ionic Concentration (mM)	Ionic Charge	Ionic Concentration × Ionic Charge
Ca^2+^	134	40	3.35	+2	13.40
Mg^2+^	405	24	16.88	+2	67.50
K^+^	0.8	39	0.02	+1	0.02
Na^+^	106	23	4.61	+1	4.61
Cl^−^	5844.7	35	166.99	−1	166.99
SO_4_^2−^	1525.7	96	15.89	−2	63.57
SeO_3_^2−^	1.3	128	0.01	−2	0.04
H_2_VO_4_^−^	0.7	117	0.01	−1	0.01
Zn^2+^	2.8	64	0.04	+2	0.18
Total (mM)					316
Ionic strength of solution (M)					0.16

**Table 2:** Sequences of the primers used for qRT-PCR analysis in this study.

Gene	Forward (5′–3′)	Reverse (5′–3′)
LDH A	GGTTACACATCCTGGGCCAT	CAGCTCAGACGAGAAGGGTG
LDH B	GTGTGATTGGAAGCGGATGC	TGCCGTACATTCCCTTCACC
PGC-1α	GGAACTGCAGGCCTAACTCC	TTGGAGCTGTTTTCTGGTGC
ERR-α	AGCCAGTCCTGACAGTCCAA	CCGGACAGCTGTACTCGATG
RXR-α	TGCGTCACTAGAAGCGTACT	GAGTAAAGATGGCGAGAGTGG
Myc	GCCCAGTGAGGATATCTGGA	ATCGCAGATGAAGCTCTGGT
MCT1	TTCAGTGCAACGACCAGTGA	AGTGGAGCCAGGGTAGAGAG
MPC1	CACAGCGGTGTCATCTGTCT	ATGGCCGCTTACTCATCTCG
MPC2	GCCTCTCAGCTGTTTCGGAT	CATCCACAAGCAAGTCCCCT
β-actin	AGCCATGTACGTAGCCATCC	CTCTCAGCTGTGGTGGTGAA
